# Moisture Distribution during Water Absorption of Ordinary Portland Cement Mortars Obtained with Low-Field Unilateral Magnetic Resonance

**DOI:** 10.3390/ma14154279

**Published:** 2021-07-31

**Authors:** Prisciliano Felipe de Jesús Cano-Barrita, Floriberto Díaz-Díaz

**Affiliations:** Instituto Politécnico Nacional, CIIDIR Unidad Oaxaca, Calle Hornos No. 1003, Col. Noche Buena, Santa Cruz Xoxocotlán, Oaxaca 71230, Mexico; fdiazd1500@alumno.ipn.mx

**Keywords:** nondestructive testing, three-magnet array, elliptical RF coil, water absorption, ordinary Portland cement, pore water, moisture content, T_2_ lifetime

## Abstract

Moisture distribution in cement-based materials is important from the durability point of view. In the present study, a portable three-magnet array with an elliptical surface radio frequency coil was used to undertake magnetic resonance measurements of moisture content in ordinary Portland cement mortar and concrete samples. Measurements along the length of the samples during capillary water absorption produced moisture content profiles that were compared with reference profiles acquired using a magnetic resonance imaging instrument. Profiles obtained with the three-magnet array were similar in shape and in penetration depth to those acquired with magnetic resonance imaging. The correlation coefficient between the moisture content measured with both techniques was r^2^ = 0.97. Similar values of saturated permeability of the mortars with identical w/c ratio were computed with the Hydrus 1D software based on the moisture content profiles. Additionally, inverse Laplace transformation of the signal decays provided the water-filled pore size distribution in saturated and unsaturated regions of the samples. The three-magnet array was successfully used to acquire nuclear magnetic resonance signal from a concrete sample, which was not possible with the magnetic resonance imaging instrument using the single-point imaging technique.

## 1. Introduction

Water plays a crucial role in reinforced concrete deterioration processes that reduce the service life of concrete structures. Knowing the moisture distribution during curing, as well as during drying or during water absorption processes, is important for the evaluation of durability properties or modeling deterioration processes of cement-based materials. This is especially true for the cover concrete that resists the penetration of aggressive agents like chloride ions or carbon dioxide [1] that diffuse through the pore water and may lead to reinforcing steel corrosion. During capillary water absorption, a simple visual inspection of the wet front movement in cement paste, mortar, or concrete samples by observation of the color change is not a reliable measure of the true depth of water penetration [2]. In addition, the initial moisture content in the sample before the water absorption process significantly affects the water absorption process and makes it difficult to interpret the sorptivity results [3]. The gravimetric method is traditionally used to determine the moisture content of cement-based materials by oven-drying samples at 105 °C until constant mass. If the moisture distribution is required, the sample is cut into slices at least 10 mm thick. With different slices along the length of the sample, the spatial moisture distribution is obtained, although with a low resolution [4]. Neutron radiography is a nondestructive technique that has been occasionally used to obtain moisture distribution profiles in drying mortar [5]. However, some corrections on the measured neutron intensity are necessary, which may be a source of uncertainties on the measured profiles. Another nondestructive technique is magnetic resonance imaging (MRI), which can be used to obtain images in 1, 2, and 3 dimensions with millimetric resolution [6]. The sample size in low-field MRI systems varies from about 13 mm up to 150 mm in diameter. Larger samples can be analyzed in expensive systems with superconducting magnets with larger bore sizes. Another alternative is the use of low-field unilateral nuclear magnetic resonance (NMR) devices [7] that have no restriction on sample size. They have been successfully used to characterize porosity and T_2_ distribution of core plugs [8], water content in wood [9], and carbonation of cement-based materials [10,11]. When studying cement-based materials by NMR, white Portland cement is normally used because it contains much less paramagnetic impurities than ordinary Portland cement. Paramagnetic impurities cause shortening of the spin–spin (T_2_) and effective spin–spin (T_2_^*^) relaxation times, which in turn cause signal loss.

In this paper, we present results from the use of a three-magnet array [8] combined with an elliptical surface RF coil [11] to undertake NMR measurements of moisture content profiles in ordinary Portland cement mortar samples. These profiles are compared with reference true water content profiles obtained using the SPI T_2_^*^ mapping MRI technique [12]. The saturated hydraulic conductivity (K_s_) was extracted from the profiles by inverse modeling with the Hydrus 1D software [13]. The three-magnet array was additionally used to explore obtaining NMR signal in an ordinary concrete sample, which contained cement and aggregates normally used in the construction industry.

## 2. Materials and Methods

### 2.1. Materials

Ordinary Portland cement (Holcim Mexico, Ciudad de Mexico, Mexico) was employed to prepare the mortar mixtures. The chemical composition of the Portland cement is given in Table 1. Silica sand (Eucomex, Estado de Mexico, Mexico) with water absorption of 0.22%, a density of 2.7, and fineness modulus of 2.86 was used as fine aggregate. Distilled water was used as mixing water and for the capillary water absorption experiments.

A prismatic concrete sample was cut from an old 0.60 w/c ratio ordinary concrete cylinder found in the lab. The cross-section area of the sample was 40 mm × 40 mm and 150 mm in length. This concrete contained ordinary Portland cement, natural river sand and gravel aggregates normally used in the local construction industry. Contrary to the silica sand used in the mortar samples, aggregates in the concrete sample contained paramagnetic impurities such as iron oxide that reduces the T_2_ and T_2_^*^ relaxation times [14].

### 2.2. Sample Preparation and Conditioning

Mortar mixtures at w/c ratios of 0.60 and 0.35, with a cement to sand ratio by mass of 1:1.5, were prepared using a planetary motion mixer. These w/c ratio values represented high and low values that are of interest in the construction industry. The mixing procedure was performed in accordance with ASTM C305 [15]. Mortar samples at 0.60 w/c ratio, measuring 50 mm in diameter and 53 mm in length, were cast in triplicate using PVC molds. Other 0.60 and 0.35 w/c ratio mortar samples, measuring 50 mm in diameter and 100 mm in length were cast in triplicate using PVC molds. All the samples were moist cured at room temperature in saturated lime water for 28 days. At the end of the moist curing period, the specimens measuring 100 mm in length were oven-dried at 50 °C until constant mass to minimize microcracking. The 0.60 w/c ratio samples measuring 50 mm in length were stored under ambient laboratory conditions for almost one year to achieve a uniform moisture content. This conditioning simulated drying of the mortar under real conditions before undertaking the water absorption experiments. The saturated porosities measured gravimetrically were 0.179 and 0.269 for the 0.35 and 0.60 w/c ratio mortar samples, respectively. The curved surface of the samples was coated with epoxy resin (West System, Bay City, MI, USA) to allow for unidirectional water absorption. The NMR signal from the epoxy resin was negligible compared with the signal of absorbed water (less than 1% of the total signal).

The same conditioning procedure described for the mortar samples was followed for the concrete sample that was oven-dried at 50 °C. Its measured gravimetric saturated porosity was 0.148.

### 2.3. Water Absorption Experiments and NMR/MRI Measurements

**Figure 1:**
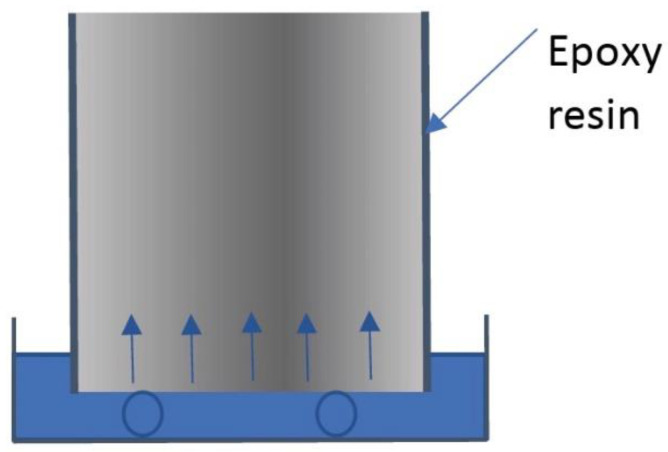
Setup for the unidirectional water absorption experiments.

The mortar samples were placed in contact with distilled water at 24 °C. Mass measurements were undertaken at various absorption times, according to the method proposed by Hall [16] and also described in the ASTM C1585 standard [17]. Figure 1 shows the setup used for the water absorption experiments.

**Figure 2:**
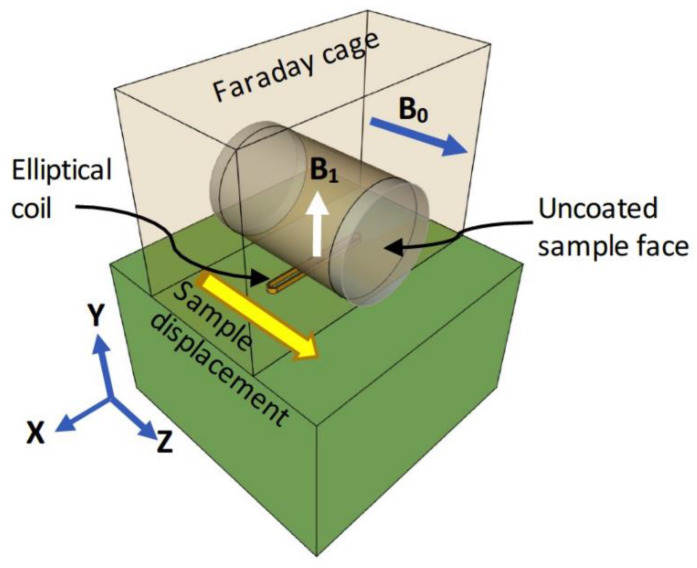
Three-magnet array [8] with the elliptical surface RF coil to excite a thin region of the sample perpendicular to the sample displacement. Reprinted with permission from ref. [11].

A three-magnet array [8] with an elliptical surface RF coil measuring 3 mm and 44 mm in minor and major internal diameters, respectively, was used with a Kea^2^ spectrometer (Magritek Limited, Wellington, New Zealand) to undertake the NMR measurements. The three-magnet array generated a sensitive spot centered at 4.64 MHz (109 mT) (see Supplementary Material in [11]). The minor axis of the coil was oriented parallel to the sample displacement direction (Figure 2). This permitted reducing the width of the excited region of the sample [11], thus a profile of signal intensity versus distance could be obtained with an adequate resolution. The sample was displaced in steps of five mm with respect to the center of the elliptical RF coil. The Carr-Purcell-Meiboom-Gill (CPMG) technique [18] was employed to obtain the transverse magnetization decay at each position along the longitudinal axis of the sample. The parameters employed for these measurements were as follows: pulse width = 12 μs, number of echoes = 32, echo time = 80 μs, repetition time = 150 ms, number of scans = 16,384, acquisition time = 63 min. To improve the signal-to-noise ratio of the NMR signal, a Faraday cage was built with aluminum foil to cover the three-magnet array system.

The CPMG decays were fitted to a biexponential decay function (Equation (1)) to obtain the T_2_ lifetime constants and their corresponding signal intensities. To obtain a moisture content profile, the signal intensity at each point position along the sample was plotted as a function of distance. The inverse Laplace transformation was additionally used to determine the T_2_ lifetime distribution [19], which is a proxy of the pore size distribution of the water-filled porosity in the sample.

(1) $$S\left(t\right)=M_{0,1}e^{\left(-\frac{t}{T_{2,1}}\right)}+M_{0,2}e^{\left(-\frac{t}{T_{2,2}}\right)}$$

where S is the signal intensity (proportional to the water content in the sample), t is time, M_0,1_ and M_0,2_ are the equilibrium sample magnetization components corresponding to the T_2,1_ and T_2,2_ lifetime constants (related to the pore sizes in the cement paste).

To compare the moisture content profiles obtained with the three-magnet array, a single point imaging (SPI) technique [20] was employed to undertake T_2_^*^ mapping measurements [12]. These measurements were required to obtain true water content profiles. A Maran DRX HF 12/50 MRI spectrometer (Oxford Instruments Ltd., Abingdon, Oxford, UK) was used, operating at 12.9 MHz. The instrument was equipped with Techron gradient amplifiers 7782 (AE Techron, Elkhart, IN, USA). Parameters employed were pulse duration = 4 μs, fifteen encoding times (t_p_) ranging from 50 to 290 μs, field of view (FOV) = 7 cm, number of scans = 512, number of points = 64, and a repetition time of 1.1 s. The total measurement time was 2.4 h. Because of the variable t_p_, the gradient step was adjusted to maintain a constant image FOV. The maximum sample length that could be tested with the MRI instrument was less than 50 mm. When the wet front was deeper than 50 mm, the SPI T_2_^*^ mapping was undertaken in two movements of the sample. In this manner, the acquired profiles overlapped 30 mm and allowed obtaining the final profile [21]. The set of acquired data at each t_p_ was Fourier transformed to obtain T_2_^*^ weighted 1D images. The image intensity at each point in the profiles was plotted versus the encoding time. The observed decays were fitted to a single exponential decay function and back extrapolated to obtain the T_2_* lifetime and its signal amplitude at each spatial point in the profile [12].

The NMR/MRI measurements on the 10 cm long mortar samples were undertaken at four days of water absorption. This time was long enough to have the movement of the wet front without significant change, about two or three millimeters per day. This condition was necessary to ensure a realistic moisture distribution profile comparable to the one acquired with the SPI T_2_^*^ mapping. Measurements on the five cm long 0.60 w/c ratio were undertaken at two days. Only one of the three samples was also tested at 12 days. This was performed to demonstrate the use of the three-magnet arrays in time-based studies.

Moisture content profiles obtained with both techniques were processed using the Hydrus 1D software (PC-Progress, Prague, Czech Republic) [22]. This software, based on Richard’s equation, was used in the inverse modeling mode to extract the saturated hydraulic conductivity (K_s_) of the mortar samples. The parameters employed for the 0.35 w/c ratio mortar samples were saturated water content θ_s_ = 0.179 m^3^/m^3^, residual water content θ_ρ_ = 0.02 m^3^/m^3^, tortuosity l (-) = −0.50, a parameter describing the pore structure of the medium n (-) = 3.25, and a scale parameter inversely proportional to the mean pore diameter α (m^−1^). For the 0.60 w/c ratio mortar samples, the parameters were θ_s_ = 0.269 m^3^/m^3^, θ_r_ = 0.02 m^3^/m^3^, l (-) = −0.50, n (-) = 3.25. In both cases, the fitting parameters were α and K_s_. Details about the Hydrus software and the meaning of parameters required by the model can be found elsewhere [13].

## 3. Results and Discussion

**Figure 3:**
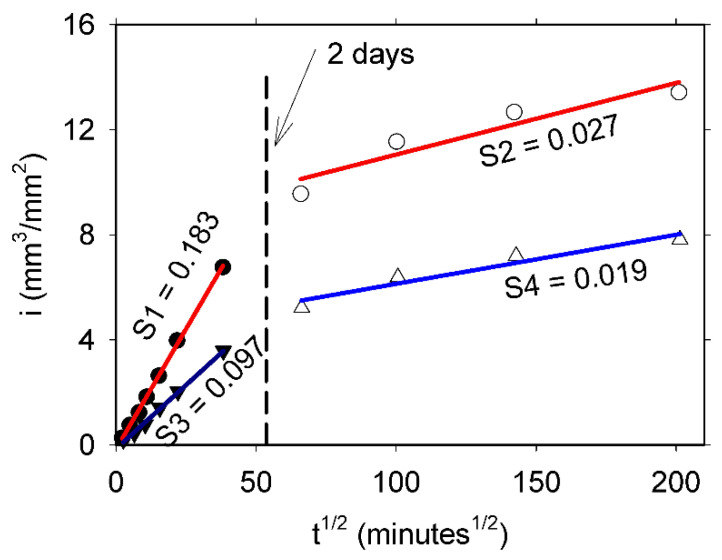
Cumulative water absorption per unit area (i) versus the square root of time for 0.60 (circles) and 0.35 (triangles) w/c ratio mortars. Primary sorptivities S1 and S3 (filled symbols) and secondary sorptivities S2 and S4 (empty symbols) are given in units of mm/min^1/2^.

Figure 3 shows a typical behavior of the water absorption process in the mortar samples, where primary and secondary sorptivities are observed. Primary sorptivity is related to the filling of large capillary pores in the hydrated cement paste, whereas the secondary sorptivity is associated with filling of gel and small capillary pores [23]. Almost two times higher primary sorptivity was exhibited by the 0.60 w/c ratio sample compared with the 0.35 w/c ratio mortar. This was expected because of the higher capillary porosity of the 0.60 w/c ratio mortar. However, in both mortars, the differences in secondary sorptivities were not as high as the differences in primary sorptivities, as found in previous research [23]. These lower secondary sorptivities, observed after two days of testing, resulted from a lower rate of water absorption. This, in turn, indicates a lower rate of penetration of the wet front and was the reason to undertake the NMR/MRI measurements after two days of water absorption.

**Figure 4:**
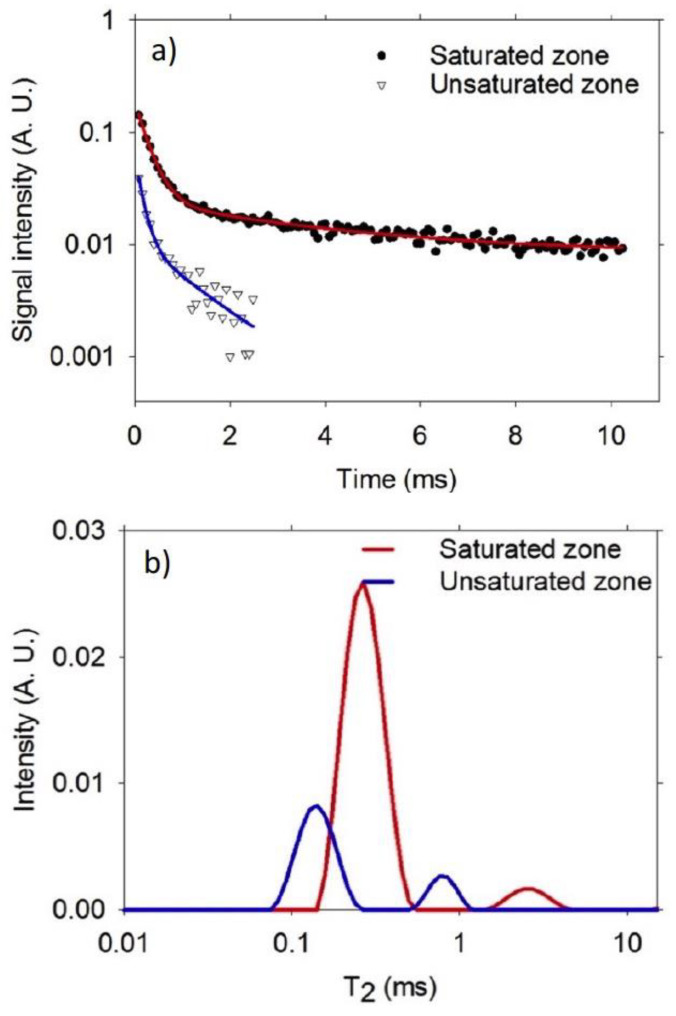
(**a**) CPMG decay in saturated and unsaturated zones of a 0.60 w/c ratio mortar sample, and (**b**) T_2_ distributions corresponding to the CPMG decays shown in (**a**).

Figure 4a shows CPMG decays in saturated and unsaturated regions of a 0.60 w/c ratio mortar sample. It was observed that there was a faster decay of the CPMG measurement undertaken in the unsaturated region, compared with the decay in the saturated region. In the unsaturated region, the smallest pores (gel and small capillary pores) were filled with water. In some cases, the high noise present in the decay made it difficult to observe the second pore population, which always has low signal intensity. On the other hand, in the saturated region, most of the pores were filled with water, including the large capillary pores. In these pores, the water molecules relaxed slower than the small capillaries and gel pores, according to Equation (2) [24].

(2) $$\frac{1\;}{T_{2}}=\;\rho \frac{S}{V}$$

where 1/T_2_ is the relaxation rate, ρ is the surface relaxivity of the pore walls, and S/V is the surface-to-volume ratio of the pore system.

The inverse Laplace transform of the decays presented in Figure 4a are given in Figure 4b. It is considered a proxy of the water-filled pore size distribution. It was assumed that at the beginning of the water absorption process, the microstructure at various positions along the sample was similar. However, as water entered, there was a pore size rearrangement in the wet region [25], as well as filling the empty pore space that contributed to the CPMG signal. A higher signal intensity was observed in the saturated zone, indicating a higher amount of water in the pore space compared with the unsaturated zone, as expected.

**Figure 5:**
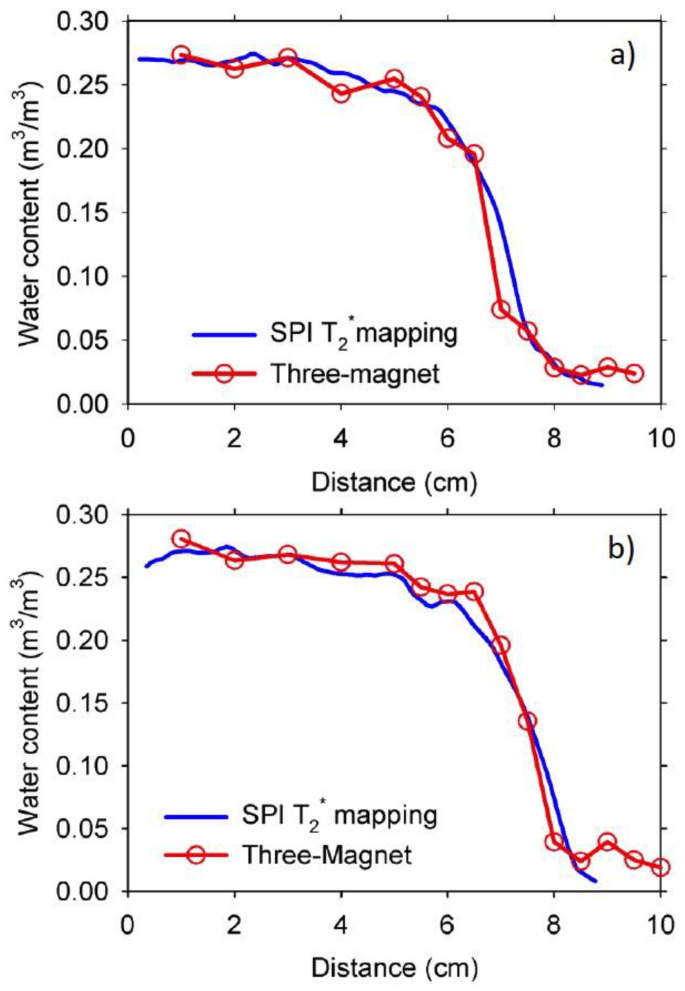
Water content distribution from SPI T_2_^*^ mapping and CPMG signal intensity for the 0.60 w/c ratio, 100 mm long duplicate mortar samples (**a**,**b**) at four days of water absorption. Profiles with the T_2_^*^ mapping were obtained by moving the sample in two steps due to the sample length restriction of the MRI instrument. The T_2_^*^ was about 171 μs.

Figure 5 shows the moisture profiles of the 0.60 w/c ratio duplicate samples with 100 mm in length. The T_2_^*^ mapping was necessary to eliminate the T_2_^*^ weighting affecting the NMR signal, which in turn would affect the shape of the profile in zones with different T_2_^*^ lifetimes [26]. In the case of the MRI measurements, the sample was displaced three centimeters from the first position to acquire a second profile that overlapped with the first one. However, the sample length was not a problem when using the three-magnet array that did not have any restriction on the sample size. The profiles obtained with both techniques were remarkably similar in regions of higher water content but did not agree completely with the lower water content region.

**Figure 6:**
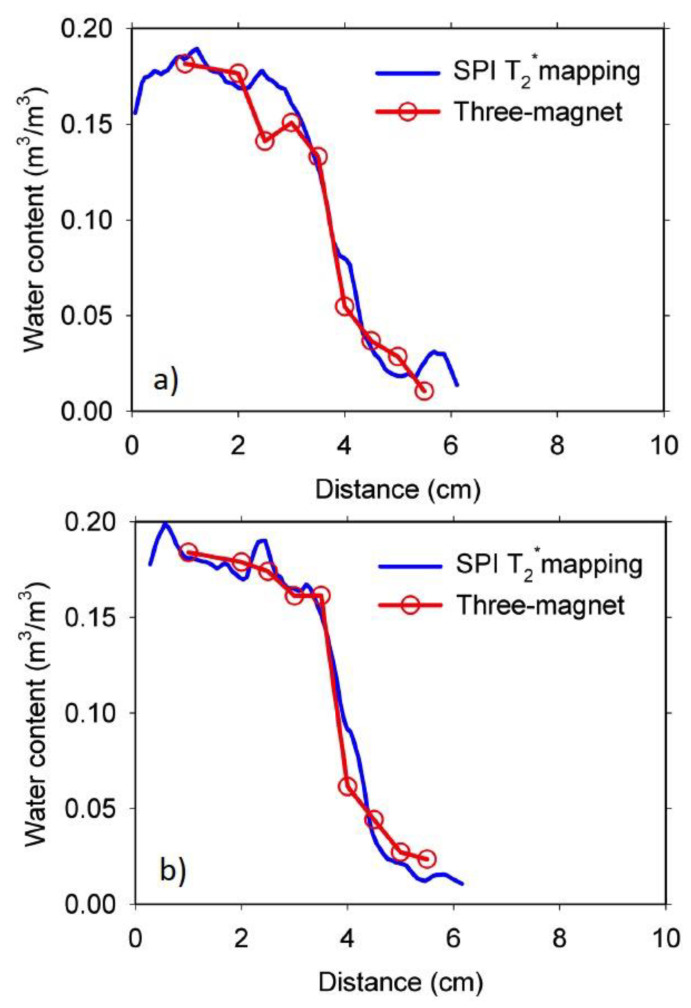
Water content distribution from SPI T_2_* mapping and CPMG signal intensity for the 0.35 w/c ratio, 100 mm long duplicate mortar samples (**a**,**b**) at four days of water absorption. The T_2_* was about 158 μs.

Results of the 0.35 w/c ratio, 100 mm duplicate mortar samples are shown in Figure 6. In spite of the lower water content compared with the 0.60 w/c ratio samples, the NMR measurements with the three-magnet array was nevertheless possible on these samples. Penetration of the wet front was reduced compared with the samples shown in Figure 5, as expected, because of the lower permeability of low w/c ratio materials.

A similar moisture content profile was obtained, compared with the profile acquired with the SPI T_2_^*^ mapping technique. Because of the inferior quality of the signal acquired at the lower moisture content region (right-hand side), fitting was not possible. Alternatively, the signal intensity of the first echo was taken.

**Figure 7:**
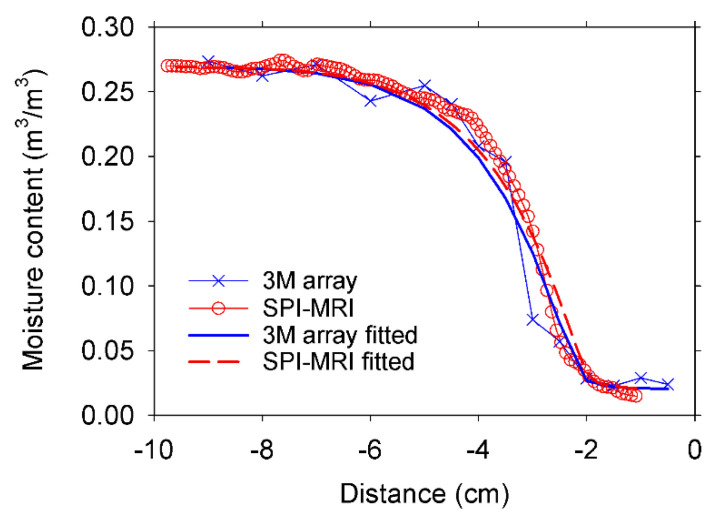
Experimental and fitted moisture distribution profiles of a 0.60 w/c ratio mortar sample. The experimental profiles were acquired with the three-magnet array and the SPI T_2_* mapping MRI technique. Fitted profiles were obtained by inverse modeling with the Hydrus software.

Figure 7 shows an example of the results obtained with the inverse modeling procedure with the Hydrus 1D software [22]. These results correspond to the 0.60 w/c ratio mortar. The r^2^ for regression of fitted versus experimental data in the profiles from all the samples was higher than 0.96. The K_s_ values obtained from the SPI profiles and the CPMG signal intensities for the four samples are given in Table 2. It showed similar K_s_ values for samples with identical w/c ratio, as expected. The mortar with a lower w/c ratio possessed a lower saturated permeability than the high w/c ratio mortar, as it is already established in the literature [26]. These results demonstrated the capability of the three-magnet array to acquire valuable data to extract transport properties of the mortars.

**Figure 8:**
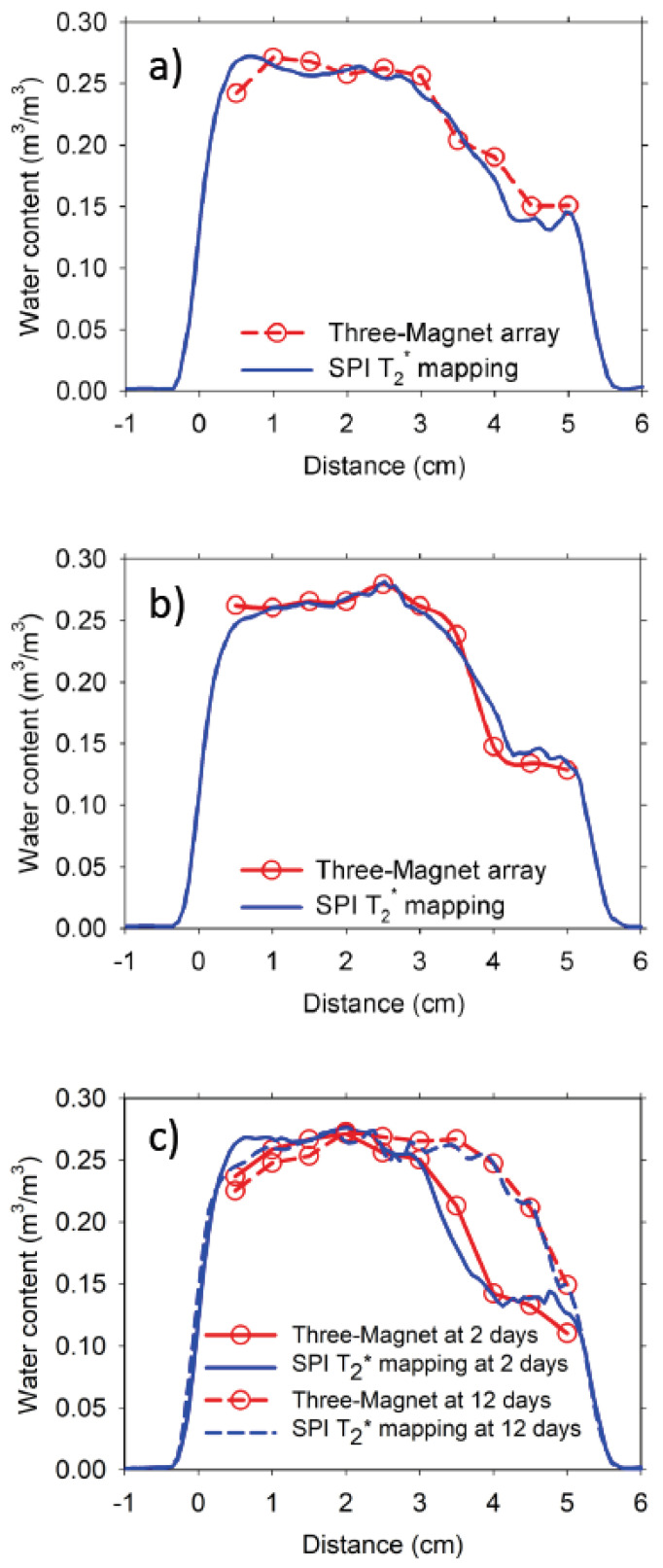
Moisture content profiles obtained with the three-magnet array with an elliptical surface RF coil, and the T_2_* mapping with the SPI technique on three 0.60 w/c ratio mortar samples, (**a**) and (**b**) at 2 days of water absorption, and (**c**) at 2 and 12 days of water absorption.

A comparison of the profiles obtained with the three-magnet array and the T_2_^*^ mapping on three identical 5 cm long samples is presented in Figure 8. The three samples showed similar moisture distribution and wet front position. These 0.60 w/c ratio mortar samples were conditioned in an ambient laboratory environment for one year before undertaking the water absorption experiments. The effect of this conditioning on the samples is shown as a relatively high moisture content on the right-hand side of Figure 8a–c. This moisture content of about 0.14 m^3^/m^3^ represents a more realistic level of moisture this mortar would have in the field, compared with oven drying at 50 °C the 10 cm samples made with the same mortar that has a moisture content of about 0.025 m^3^/m^3^ at the drier zone (see the right-hand side of Figure 5a,b). Additionally, the three-magnet array may be used to undertake time-based studies, as shown in Figure 8c (profiles obtained at 2 and 12 days of water absorption).

**Figure 9:**
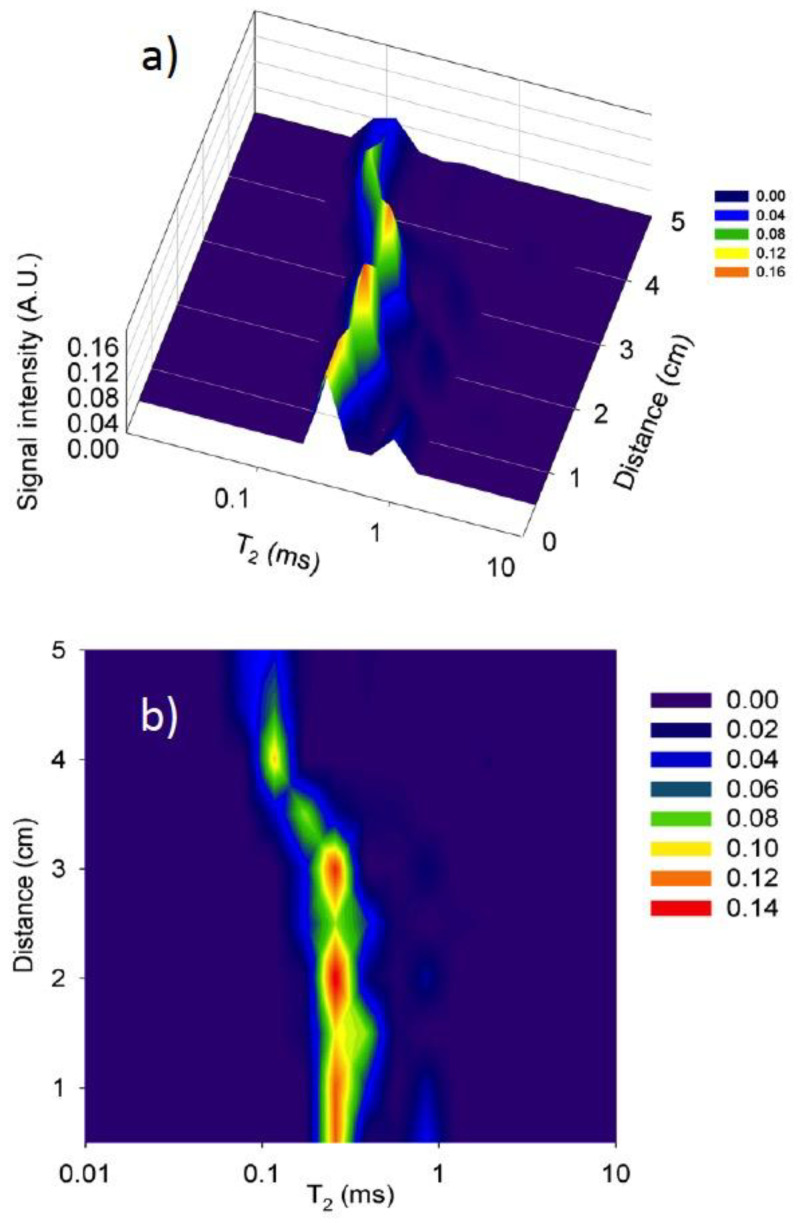
T_2_ lifetime distributions along the length of a 0.60 w/c ratio mortar sample obtained by inverse Laplace transformation of the CPMG decays from the mortar sample shown in Figure 8a, at 2 days of water absorption: (**a**) 3D view and (**b**) 2D view.

Figure 9 shows the ILT of the CPMG decays obtained along the length of one of the samples shown in Figure 8. There were two pore-populations with longer T_2_ values starting at the absorbing face and continuing along the region with high water content. This indicated that most of the pore space (gel and capillary) is filled with water. In some cases, only one pore population was observed (e.g., 1.5 cm and 2.5 cm). This was because of the noise in the signal that made it difficult to resolve the second pore population. In contrast, the unsaturated zone exhibits mainly one pore population with shorter T_2_ values comprising mainly small pores (gel and small capillaries) filled with water during conditioning at ambient temperature and relative humidity.

**Figure 10:**
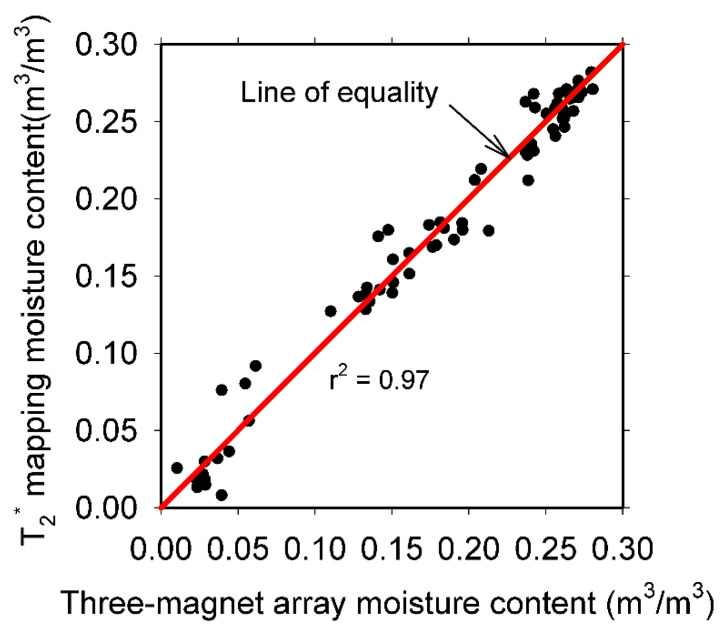
Three-magnet array moisture content versus the moisture content obtained with the T_2_* mapping of all the mortar samples tested.

The correlation between the moisture content obtained at the same position in the mortar samples, using both techniques is shown in Figure 10. A high determination coefficient was obtained (r^2^ = 0.97), indicating the suitability of the three-magnet array to acquire relative moisture content values that are comparable to those obtained with magnetic resonance imaging techniques like SPI T_2_^*^ mapping.

The three-magnet array does not have a restriction on the sample size, compared with the MRI system used for imaging the moisture content profiles. In addition, its nondestructive nature is advantageous compared with destructive techniques like slicing the sample for oven drying [4], which requires preparing several samples to be tested in addition to a lower spatial resolution. The three-magnet array with the elliptical RF coil can even be used in spatial movements of 0.25 cm, which will increase the spatial resolution as demonstrated in previous research on carbonation of mortars [11]. These measurements were not aimed for water absorption times when the wet front position changed rapidly during the NMR measurements. This is because the shape of the signal intensity profiles (evaporable water content) would be unreliable.

The T_2_* of water in the mortars ranged from 158 to 171 μs for the 0.35 and 0.60 w/c ratio samples, respectively. In contrast, the T_2_* of water in the concrete sample was about 20 μs. This sample contained normal weight aggregate (river sand and gravel) used in commercial concrete production. These aggregates typically contain paramagnetic impurities, mainly iron oxide, in a range of about 1% to even 10%. This iron content, additional to the iron present in the ordinary Portland cement, enhances the relaxation of the protons, therefore reducing the T_2_* and T_2_ lifetimes. It is known that the relaxation rate 1/T_2_ is proportional to the concentration of paramagnetic impurities, like Fe_2_O_3_ [14]. The very short T_2_* lifetime in the concrete sample precluded acquisition of an SPI profile even with the shortest encoding time (t_p_ = 50 μs) that could be used in the MRI system.

**Figure 11:**
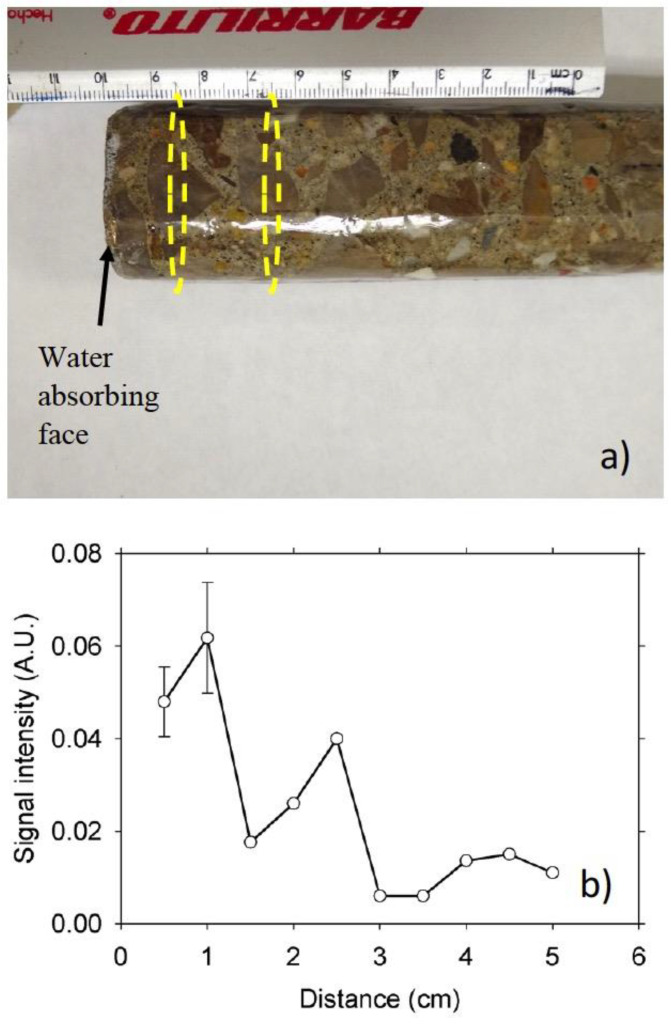
(**a**) Photograph of the 0.60 w/c ratio concrete sample. The dotted ellipses show the approximate position of the RF coil during the NMR measurements at positions 1.5 and 3.5 cm where the presence of coarse aggregates reduced the signal intensity, and (**b**) CPMG signal intensity after five days of water absorption. The sample error bars represent the uncertainty in the estimation of the intensity.

However, the three-magnet array was successfully used to undertake CPMG measurements in the concrete sample. Figure 11a is a photograph of the concrete sample used for the NMR measurements, clearly showing the gravel’s coarse aggregates. Figure 11b is the CPMG signal intensity (moisture content) profile along the length of the sample. The dips observed in the profile (e.g., at 1.5 and 3.5 cm) corresponded to the presence of coarse aggregates that reduced the intensity of the NMR signal. In the low signal intensity points (≥3 cm), an average of the first two echoes of the CPMG decay was taken because of the low signal-to-noise ratio that precluded fitting of the data to an exponential decay function. The CPMG profiles in concrete samples could be improved by undertaking a second or a third measurement at the same distances but in another line in the rotated sample (90° or 180°). An average of the signal intensities at each point position can be taken. This will undoubtedly increase the measurement time for each sample but would produce a better profile.

The ability to obtain moisture content profiles with the three-magnet array in cement-based materials will permit calculation of the transport properties needed in models for prediction of the coupled ion-moisture transport [27], chlorides concentration at the reinforcement level in concrete subject to wick action [28], or other deleterious processes [29] to help predict the service life of concrete structures.

Future research will focus on NMR measurements with the three-magnet array and the elliptical surface RF coil to monitor moisture distribution in processes such as drying of cement-based materials or other porous materials like wood, rocks, or food. It will also be possible to monitor microstructural changes in samples as a function of position or as a function of time.

## 4. Conclusions

This paper has demonstrated the use of a three-magnet array with an elliptical surface RF coil to determine moisture content at various positions along mortar and concrete samples during water absorption experiments. Based on this study, the following conclusions are drawn:

- A three-magnet array with an elliptical surface RF coil operating at a frequency of 4.64 MHz, allows the determination of the NMR signal along mortar and concrete samples containing ordinary materials and with no limitation on the sample size.
- In the concrete sample, the very short NMR signal lifetime (T_2_* ≈ 20 μs) caused by paramagnetic impurities in the material precluded the acquisition of an MRI profile.
- There is a linear relationship between the signal intensities (moisture content) obtained with the three-magnet array and the corresponding signal intensities from MRI profiles taken as a reference (r^2^ = 0.97).
- Similar values of saturated permeability (K_s_) of mortar samples with identical w/c ratios were obtained from profiles acquired by the two techniques outlined in this paper, demonstrating the feasibility of the three-magnet array with an elliptical surface RF coil to acquire valuable data for transport properties determination of cement-based materials.

## Figures and Tables

**Table 1:** Chemical composition of the Portland cement.

Oxide	Weight (%)
SiO_2_	21.07
Al_2_O_3_	3.69
Fe_2_O_3_	4.50
CaO	61.93
MgO	1.83
Na_2_O	0.09
K_2_O	0.30
SO_3_	2.54
LOI	4.38

**Table 2:** K_s_ values of the 0.60 and 0.35 w/c ratio mortars, obtained from inverse modeling using the Hydrus software.

Sample	w/c Ratio	K_s_ (m/s) SPI-MRI	K_s_ (m/s) Three-Magnet Array
1	0.60	7.63 × 10^−12^	7.33 × 10^−12^
2	0.60	7.97 × 10^−12^	7.88 × 10^−12^
3	0.35	1.37 × 10^−12^	1.36 × 10^−12^
4	0.35	1.55 × 10^−12^	1.35 × 10^−12^

## Data Availability

Data is contained within the article.
